# Dark-white matter sign in uncontrolled diabetes: a radiologic marker of critical metabolic injury

**DOI:** 10.1055/s-0046-1825763

**Published:** 2026-07-28

**Authors:** Rodolfo E. Arce, Kevin J. Abrams, Leonardo Furtado Freitas

**Affiliations:** 1 Florida International University, Herbert Wertheim College of Medicine, Department of Internal Medicine, Miami FL, United States.; 2 Baptist Health South Florida, Radiology Associates of South Florida, Division of Clinical Neuroradiology, Department of Radiology, Miami FL, United States.; 3 Florida International University, Herbert Wertheim College of Medicine, Miami FL, United States.

**Figure 1:**
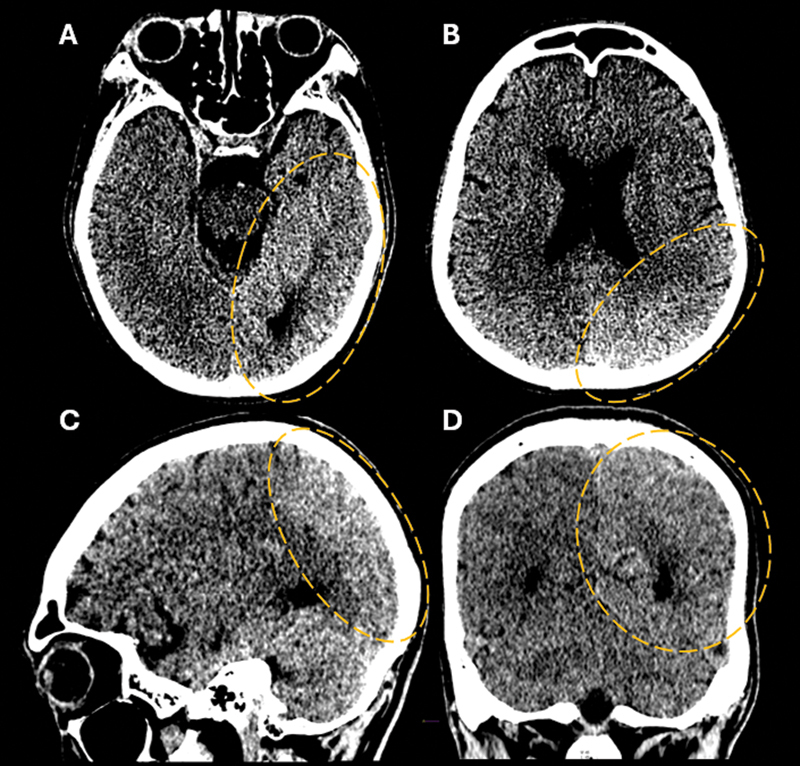
Noncontrast head computed tomography (CT). ( **A,B** ) Axial, ( **C** ) sagittal, and ( **D** ) coronal views. Cortico subcortical swelling and hyperdensity in the left parieto-temporo-occipital lobe (orange dashed circles).

**Figure 2:**
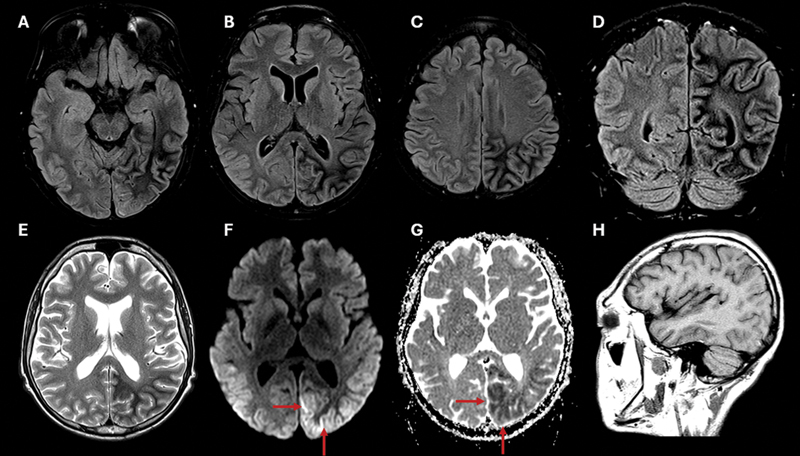
Noncontrast brain magnetic resonance imaging (MRI). ( **A–D** ) Fluid-attenuated inversion recovery (FLAIR) sequences, ( **E** ) T2-weighted image, ( **F** ) diffusion-weighted imaging (DWI), and ( **G** ) apparent diffusion coefficient (ADC) map. The cortical subcortical involvement demonstrated prominent T2/FLAIR hypointensity (“dark white matter”), accompanied by focal restricted diffusion (red arrows) due to superimposed cytotoxic edema. No significant abnormalities were observed on ( **H** ) the T1-weighted sequence.

A 33-year-old man with type-2 diabetes mellitus presented with severe altered mental status in the setting of recurrent diabetic ketoacidosis and poor insulin adherence, during which an unwitnessed hyperglycemia-related seizure could not be excluded. Laboratory studies confirmed marked hyperglycemia (483 mg/dL), markedly elevated hemoglobin A1c (13%), high anion gap acidosis, and hyperosmolar dehydration. Computed tomography (CT) scans showed a hyperdense cortico subcortical region ( Figure 1 ), while magnetic resonance imaging (MRI) demonstrated T2/fluid-attenuated inversion recovery (FLAIR) hypointensity ( Figure 2 ), also known as “dark white matter”, a pattern linked to hyperglycemia-related seizures. Proposed mechanisms include altered oxygen extraction, perfusion changes, excitotoxicity, and free radical–induced T2 shortening. Although these findings may resolve after metabolic correction, they do not necessarily indicate full tissue recovery, underscoring the importance of recognizing this transient imaging pattern. 1 2
